# Secular trends in the prevalence of meeting 24-hour movement guidelines among U.S. adolescents: evidence from NHANES 2007–2016

**DOI:** 10.3389/fpubh.2024.1362718

**Published:** 2024-04-03

**Authors:** Xue-qing Liu, Mei-ling Liu, Zhuo-wen Wu, Jing-hong Liang

**Affiliations:** ^1^School of History, Qingdao University, Qingdao, China; ^2^Department of Maternal and Child Health, School of Public Health, Sun Yat-sen University, Guangzhou, China

**Keywords:** trends, prevalence, adolescents, 24-hour movement, NHANES

## Abstract

**Background:**

The 24-Hour Movement Guidelines (24-HMG) recommend a balanced combination of physical activity (PA), sedentary behavior (SB) and sleep (SLP) for optimal health. However, there is limited understanding of how well U.S. adolescents adhere to these guidelines. This study aims to analyze the prevalence trends of meeting the 24-HMG among a nationally representative sample of U.S. general adolescents.

**Methods:**

The study included 2,273 adolescents (55.3% boys) aged 16–19 who participated in the National Health and Nutrition Examination Surveys (NHANES) from 2007 to 2016. The researchers categorized the adolescents based on whether they met various PA, SB, and SLP recommendations, as well as different combinations of these recommendations, separately for boys and girls. The prevalence rate, weighted by survey data, was calculated along with a 95% confidence interval (CI) to assess the changes in meeting the 24-HMG among U.S. adolescents across different survey years and sociodemographic subgroups.

**Results:**

In the 2015–2016 cycle, approximately 6.3% of adolescents did not meet any of the three recommendations, while only 19.2% of adolescents achieved all three guidelines. Compliance with PA and SB recommendations among adolescents has decreased over time, from 72.5% (65.9% to 79.2%) to 64.2% (57.4% to 70.9%) for PA, and from 59.0% (49.6% to 68.4%) to 46.6% (37.8% to 55.5%) for SB, respectively, from 2007–2008 cycle to 2015–2016 cycle. Boys exhibited more favorable patterns in meeting different sets of recommendations compared to girls (*p*-value <0.001). This includes meeting both PA and SB guidelines (15.5% for boys and 11.1% for girls) and meeting both PA and SLP guidelines (19.5% for boys and 15.7% for girls). The level of parental education was found to have effect on meeting all three guidelines (*P*_trend_ < 0.05).

**Conclusion:**

We analyzed ten consecutive years of representative NHANES data to evaluate the prevalence meeting 24-HMG and found that the proportion of adolescents aged 16–19 in the U.S. who adhered to all three movement guidelines simultaneously has consistently remained low throughout each survey cycle. Notably, there has been a significant decline in the proportion of adolescents meeting the SB guideline.

## Introduction

Low levels of physical activity (PA), high amounts of sedentary behavior (SB), and inadequate sleep (SLP) have been widely reported, particularly in high-income countries like the U.S. (1). According to the Youth Risk Behavior Survey, there has been a persistently low prevalence of high school students engaging in at least 60 min of PA per day throughout the week from 2019 to 2021 (2). During the COVID-19 pandemic, U.S. children were found to spend approximately 90 min sitting for school-related activities and over 8 h sitting for leisure activities each day (3). Furthermore, a study involving individuals from the Netherlands, UK, and U.S. highlighted that more than half of adolescents aged 14–17 years obtained less than the recommended 8–10 h of SLP per night (4).

The 24-Hour Movement Guidelines (24-HMG) have been recently released in several countries including Australia (5), Canada (6), and New Zealand (7). These guidelines focus on integrating three movement behaviors – PA, SB and SLP – throughout the day to promote a healthy balance. Adolescence is a critical period for physical and mental development and for acquiring resources regarding the foundation for later life health and wellbeing (8). Adolescents are highly malleable and their behavioral habits are easily affected (9). If they developed healthy behaviors during this period, especially appropriate 24-hour movement behaviors, can may effectively promote wellness. Research has shown that for children and adolescents, it is more beneficial for their overall physical and psychological health to meet all three movement behavior recommendations rather than just meeting one recommendation alone (10–12). Adolescents who achieve multiple recommended health behaviors have been found to have lower waist circumference compared to those who only meet one recommendation or do not adhere to any of these recommendations. This suggests that combinations of health behaviors may interact to produce beneficial effects (13). Similarly, another study found that children who met all three recommendations or both screen time and SLP recommendations demonstrated superior global cognition. This highlights the importance of limiting recreational screen time and promoting healthy SLP habits to enhance cognition in children (14).

Since the adoption of the 24-HMG, there has been a gradual increase in the number of studies focusing on individuals meeting three guidelines, although this area of research is still in its early stages. To the best of our knowledge, most previous studies have only examined whether an individual meets a single guideline. For instance, one study found that from 2007 to 2018, the age-standardized prevalence of U.S. adults meeting PA guidelines increased from 19.4% to 25.3% for urban residents and from 13.3% to 19.6% for rural residents, but the overall level of compliance remained relatively low (15). Other studies have also examined the combinations of three movement behaviors, but they too have certain limitations. For example, some studies have evaluated compliance with meeting combinations involving any two out of the three guidelines (16). Others have focused primarily on the prevalence of meeting 24-HMG among special populations (17–19), which restricts the generalization to the wider healthy population. Previous studies (14, 20, 21) have failed to assess the long-term trends for compliance, which has resulted in a lack of an overall understanding of the prevalence over decades. This lack of understanding hinders the development of effective prevention and control strategies. Additionally, due to the good representation and wide acceptance of the National Health and Nutrition Examination Surveys (NHANES) database, an increasing number of studies have utilized the database to explore the association between behavioral factors and health (22). Therefore, given the current lack of data on the latest trends and long-term patterns of U.S. adolescents meeting these guidelines and strength of NHANES database, this study aims to provide representative estimate of the secular prevalence trend using data from NHANES conducted between 2007 and 2016.

## Methods

### Study participants

The NHANES is an annual survey conducted by the National Center for Health Statistics (NCHS) to evaluate the health and nutritional status of non-institutionalized U.S. civilians (23). Participants were selected using a complex, stratified, and multistage probability sampling design. The surveys include questions about demographics, socioeconomic status, diet, and health through interviews, as well as medical and physiological measurements obtained through physical examinations. The NHANES program was supported and approved by the Ethics Review Board of NCHS, and written informed consent was obtained from all participants.

The analysis began with a total of 114,039 participants from the NHANES survey cycles conducted between 2007 and 2016. Initially, we focused on individuals aged 16–19 years, resulting in a study population of 82,986. We then excluded participants who did not have survey-weight data (*n* = 1,695), those with missing outcome variables (*n* = 21,557), and those with unavailable information on demographic variables (*n* = 5,528). Finally, the final analysis included a total of 2,273 adolescents.

### Measurements

Exposures of interest in the study were PA, SB, and SLP. In the NHANES dataset, information on PA was collected using a PA questionnaire based on the Global Physical Activity Questionnaire (24). The questionnaire included self-reported questions about the number of days and minutes individuals engaged in moderate to vigorous physical activity (MVPA) per week, taking into account the duration and frequency of each type of PA. SB was assessed by asking participants about the amount of time they typically spend sitting or reclining during a typical day, including time spent at work, home, or school (such as sitting at a desk, sitting with friends, traveling in a car, bus, or train, reading, playing cards, watching television, or using a computer), excluding time spent sleeping. SLP duration was self-reported by participating adolescents and measured as the number of hours of SLP per night.

According to the Canadian 24-HMG (25), adolescents who reported accumulating at least 60 min of MVPA per day (for those aged 16–17 years) or at least 150 min/day of MVPA (for those aged 18–19 years), no more than 2 h/day of recreational screen time (for those aged 16–17 years) or no more than 3 h/day (for those aged 18–19 years), and getting an uninterrupted 8–10 h/night of SLP (for adolescents aged 16–17 years) or an uninterrupted 7–9 h/night of SLP (for adolescents aged 18–19 years), respectively, were considered as meeting individual recommendations (meeting PA, meeting SB, or meeting SLP). The recommendations for PA and SLP are consistent with those established by the World Health Organization and the National Sleep Foundation (26, 27). Additionally, meeting the 24-HMG recommendations was assessed as a continuous variable (General combinations: meeting none, meeting only one out of three, meeting any two out of three, meeting all three) and as a categorical variable (Specific combinations: only meeting PA, only meeting SB, only meeting SLP, meeting PA and SB, meeting PA and SLP, meeting SB and SLP).

### Covariates

Potential confounders as follow were screened for statistical analysis based on prior researches (22, 28). We obtained information from NHANES database including age (numeric), gender (boy or girl), race/ethnicity (non-Hispanic White, non-Hispanic Black, Mexican American, and other races (including multi-racial and other Hispanic)), parental education level (less than 9th grade, 9–12th grade (including 12th grade with no diploma), and more than 12th grade), total energy intake (quartile), poverty to income ratio (PIR, which is used to assess a person whether lives with household income below the federal poverty level, and sorted into below poverty (< 1.0) or above poverty (≥ 1.0)), body mass index (BMI, which is calculated as weight in kilograms divided by height in meters squared).

### Statistical analysis

Given the complex sampling designs and sampling weights in our analyses, we incorporated appropriate sample weights that accounted for differential non-response and/or non-coverage rates, as well as adjustments for oversampling of specific groups, according to the NHANES Analysis and Reporting Guidelines (28–30). Categorical variables were described with sample counts (*n*) and percentage (%), while continuous variables were described with mean ± standard error (SE). We used chi-square tests for categorical variables and *t*-tests for numerical variables, respectively, to estimate sample characteristics differences by gender, race/ethnicity, and survey years. The survey-weighted prevalence rate and 95% confidence interval (CI) for meeting 24-HMG including individual recommendations, general combinations, and specific combinations are calculated overall and further stratified by survey years. Test for trend was conducted using R package “compareGroups” to examine tendency of prevalence from 2007 to 2016, and for number of meeting three recommendations. Additionally, subgroup analyses were carried out based on the general combinations, individual recommendations, and specific combinations, respectively. For all statistical tests, the bilateral *p*-value being less than 0.05 indicated statistical significance. All analyses were conducted by R language (x64 version 4.1.0; R Foundation for Statistical Computing).

## Results

### Characteristics of study participants

According to Table 1, the survey collected data from a total of 2,273 participating adolescents, representing approximately 67,434,588 U.S. adolescents. The mean age of the participants was 17.44 (SE = 0.05) years. Out of the total participants, 55.3% (*n* = 1,256) were boys and 30.4% (*n* = 690) were non-Hispanic White. Additionally, 707 adolescents lived in households with income below the poverty threshold, and 1,897 adolescents had parents with an education level between 9th and 12th grade, including 12th grade with no diploma. The mean BMI of all participants was 25.03 (SE = 0.18) kg/m^2^. In terms of 24-hour movement behaviors, on average, participants reported engaging in 1280.30 (SE = 40.03) minutes of MVPA per week, 414.57 (SE = 6.98) minutes of SB per day, and 7.61 (SE = 0.04) hours of SLP per night.

**Table 1:** Demographic characteristics variables of U.S. adolescents aged 16–19 years stratified by gender, NHANES 2007–2016 (*n* = 2,273).

Characteristics	Estimate U.S. population	Total participants	Gender		*p*-value
			Boys	Girls	
**No. of participants**	67,434,588	2,273 (100.0)	1,256 (55.3)	1,017 (44.7)	–
**Age, years**	–	17.44 ± 0.05	17.54 ± 0.06	17.31 ± 0.06	**<0.05**
**Race/ethnicity**					
Non-Hispanic White	39,661,366	690 (30.4)	388 (30.9)	302 (29.7)	0.49
Non-Hispanic Black	9,631,722	566 (24.9)	318 (25.3)	248 (24.4)	
Mexican American	9,044,757	506 (22.3)	283 (22.5)	223 (21.9)	
Other races (including multi-racial, other Hispanic)	9,096,743	511 (22.5)	267 (21.3)	244 (24.0)	
**BMI, kg/m** ^**2**^	–	25.03 ± 0.18	25.11 ± 0.23	24.94 ± 0.29	0.65
**PIR**	–	2.45 ± 0.09	2.48 ± 0.10	2.40 ± 0.12	0.52
Below poverty (<1.0)	15,907,136	707 (31.1)	374 (29.8)	333 (32.7)	0.14
Above poverty (≥1.0)	51,527,451	1,566 (68.9)	882 (70.2)	684 (67.3)	
**Parental education level**					
Less than 9th grade	1,901,955	71 (3.1)	45 (3.6)	26 (2.6)	0.27
9–12th grade (including 12th grade with no diploma)	55,344,165	1,897 (83.5)	1,050 (83.6)	847 (83.3)	
More than 12th grade	10,188,467	305 (13.4)	161 (12.8)	144 (14.2)	
**Total energy intake, kcal**	–	2235.18 ± 29.39	2548.33 ± 43.21	1855.41 ± 40.74	**<0.001**
Quartile 1 (76, 1505]	16,626,939	563 (24.8)	183 (14.2)	380 (37.3)	**<0.001**
Quartile 2 (1505, 2014]	15,872,385	568 (25.0)	290 (21.4)	278 (26.2)	
Quartile 3 (2014, 2692]	17,927,290	576 (25.3)	340 (28.4)	236 (24.4)	
Quartile 4 (2692, 10436]	17,007,973	566 (24.9)	443 (36.0)	123 (12.1)	
**MVPA, min/week**	–	1280.30 ± 40.03	1600.24 ± 69.30	892.30 ± 48.95	**<0.001**
**SB, min/day**	–	414.57 ± 6.98	399.71 ± 8.54	432.60 ± 9.86	**<0.05**
**SLP time, h/night**	–	7.61 ± 0.04	7.67 ± 0.05	7.55 ± 0.06	0.13
**Individual recommendations** ^*^					
Meeting PA	47,283,726	1,559 (68.6)	983 (78.8)	576 (59.6)	**<0.001**
Meeting SB	35,709,776	1,185 (52.1)	710 (55.8)	475 (49.5)	0.08
Meeting SLP	40,553,337	1,293 (56.9)	745 (62.9)	548 (56.8)	0.06
**General combinations**					
Meeting none	4,349,612	165 (7.3)	52 (3.5)	113 (10.0)	**<0.001**
Meeting only one out of three	19,144,891	683 (30.1)	316 (24.7)	367 (32.8)	
Meeting any two out of three	27,418,306	921 (40.5)	542 (42.4)	379 (38.5)	
Meeting all three	16,521,779	504 (22.2)	346 (29.3)	158 (18.7)	
**Specific combinations**					
Only meeting PA	9,654,649	342 (15.1)	186 (14.5)	156 (14.1)	**<0.001**
Only meeting SB	3,760,613	124 (5.5)	46 (3.6)	78 (8.0)	
Only meeting SLP	5,729,629	217 (9.6)	84 (6.7)	133 (10.7)	
Meeting PA and SB	9,116,376	349 (15.4)	227 (15.5)	122 (11.1)	
Meeting PA and SLP	11,990,922	364 (16.0)	224 (19.5)	140 (15.7)	
Meeting SB and SLP	6,311,007	208 (9.2)	91 (7.4)	117 (11.7)	
**Survey years**					
2007–2008	13,862,963	429 (18.9)	243 (20.1)	186 (21.1)	0.60
2009–2010	12,375,849	490 (21.6)	294 (18.5)	196 (18.2)	
2011–2012	13,911,006	423 (18.6)	233 (21.8)	190 (19.2)	
2013–2014	14,347,859	497 (21.9)	261 (22.0)	236 (20.4)	
2015–2016	12,936,911	434 (19.1)	225 (17.6)	209 (21.2)	

Data are presented as counts (*n*) and percentage (%) for categorical variables or mean ± SE for numerical variables. *p*-values were calculated using *t*-test or Mann–Whitney U test (numerical variables) or using chi-square test or Fisher exact test (categorical variables) to represent the differences between boys and girls. *p*-values presented with bold valued were statistically significant. ^*^ For adolescents aged 16–17 years: PA, an accumulation of at least 60 min/day of MVPA; SB, no more than 2 h/day of recreational screen time; SLP, uninterrupted 8 to 10 h/night of sleep. For adolescents aged 18–19 years: PA, an accumulation of at least 150 min/day of MVPA; SB, no more than 3 h/day of recreational screen time; SLP, uninterrupted 7 to 9 h/night of sleep. BMI, body mass index; MVPA, moderate to vigorous physical activity; NHANES, National Health and Nutrition Examination Survey; PA, physical activity; PIR, poverty to income ratio; SB, sedentary behavior; SE, standard error; SLP, sleep.

### Prevalence of U.S. adolescents meeting the 24-hour movement guidelines

**Figure 1:**
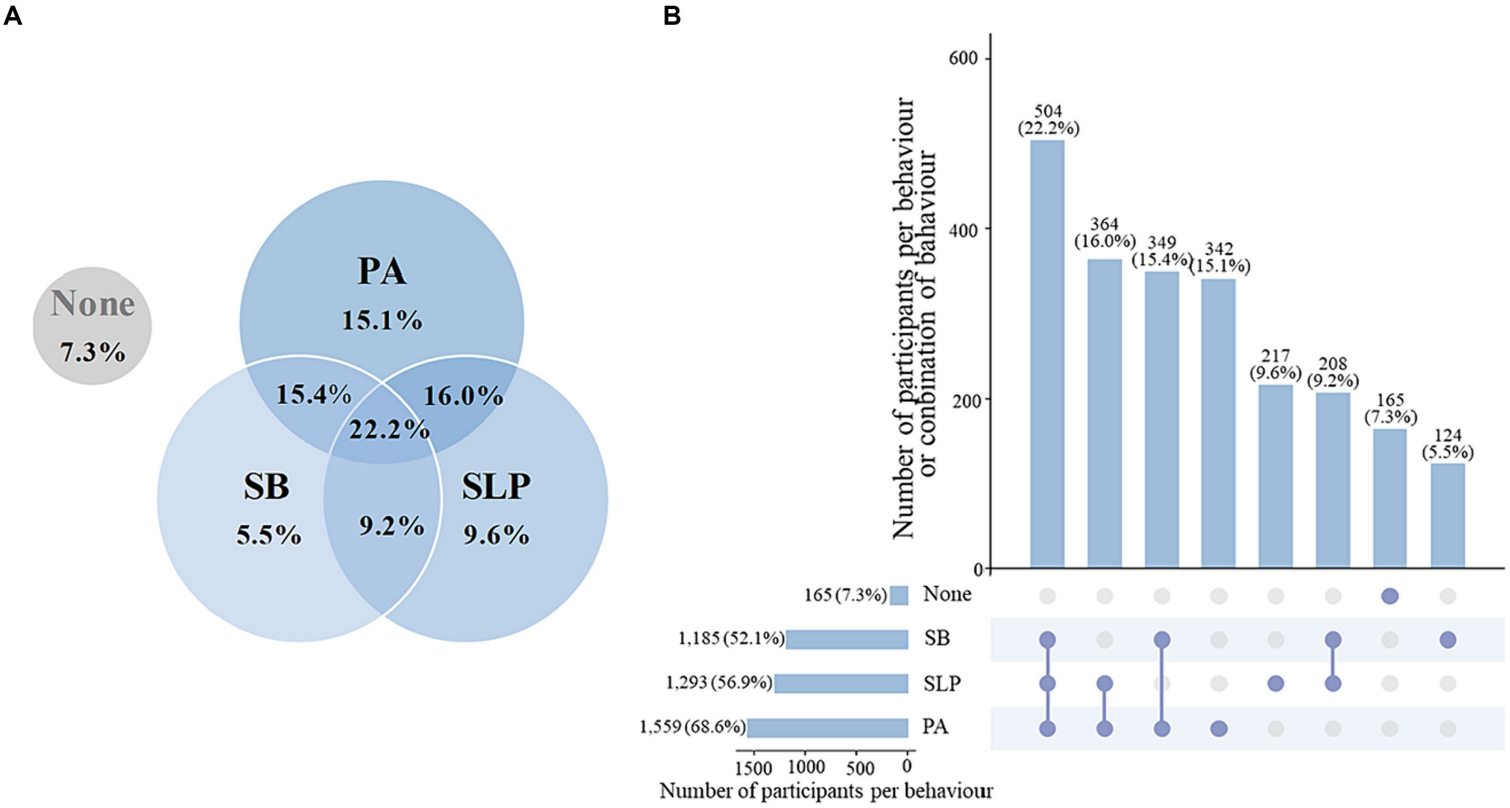
Proportions of U.S. adolescents meeting 24-hour movement guidelines recommendations, NHANES 2007–2016 (*n* = 2,273). **(A)** Venn diagram shows un-weighted proportions of various combinations of meeting PA, SB and SLP. For example, the overlap of PA, SB, and SLP means that the proportions of U.S. adolescents meeting all three recommendations simultaneously is 22.2%. **(B)** Upset diagram shows the numbers and proportions of various combinations of meeting PA, SB and SLP, as well as the graph in the lower left corner shows the numbers and proportions of adolescents meeting individual recommendations. For example, there are three blue dots representing PA, SB and SLP below the first column, which means that 504 (22.2%) U.S. adolescents meet the recommendations for PA, SB and SLP synchronously. NHANES, National Health and Nutrition Examination Survey; PA, physical activity; SB, sedentary behavior; SLP, sleep.

Table 2 displays the prevalence of meeting 24-HMG among total participants, including individual recommendations, general combinations, and specific combinations. From 2007 to 2016, the percentages of adolescents meeting the PA, SB, and SLP recommendations were 70.1% (95% CI: 63.2% to 77.0%, *n* = 1,559), 53.0% (95% CI: 46.3% to 59.6%, *n* = 1,185), and 60.1% (95% CI: 53.0% to 67.3%, *n* = 1,293), respectively. Regarding general combinations indicating the number of recommendations met, 6.5% (95% CI: 5.1% to 7.8%, *n* = 165) of adolescents met none of the three recommendations, 28.4% (95% CI: 25.0% to 31.8%, *n* = 683) met one recommendation alone, 40.7% (95% CI: 35.6% to 45.7%, *n* = 921) met any two out of the three recommendations, and 24.5% (95% CI: 19.8% to 29.2%, *n* = 504) met all three recommendations. Among adolescents who met two of the three recommendations, the majority (*n* = 364) adhered to recommendations concerning the combination of PA and SLP. The overall distribution of U.S. adolescents meeting the 24-HMG is depicted in Figure 1.

**Table 2:** Prevalence and change of meeting the 24-hour movement guidelines among U.S. adolescents aged 16–19 years, stratified by survey years, NHANES, 2007–2016 (*n* = 2,273).

Meeting recommendations	Total participants		Survey years, prevalence (95% CI)					Change, prevalence (95% CI)	*P*_trend_
	*n*	Prevalence (95% CI)	2007–2008 (*n* = 429)	2009–2010 (*n* = 490)	2011–2012 (*n* = 423)	2013–2014 (*n* = 497)	2015–2016 (*n* = 434)	2015–2016 *vs* 2007–2008	
**Individual recommendations** ^*^									
Meeting PA	1,559	70.1 (63.2,77.0)	72.5 (65.9,79.2)	68.4 (64.2,72.6)	78.3 (70.4,86.1)	66.7 (60.2,73.3)	64.2 (57.4,70.9)	−8.4 (−17.8,1.1)	0.365
Meeting SB	1,185	53.0 (46.3,59.6)	59.0 (49.6,68.4)	59.5 (51.4,67.6)	58.3 (46.4,70.2)	41.9 (32.3,51.6)	46.6 (37.8,55.5)	−12.4 (−25.3,0.5)	0.093
Meeting SLP	1,293	60.1 (53.0,67.3)	55.9 (50.7,61.1)	56.0 (49.9,62.1)	67.1 (59.2,75.1)	60.7 (54.0,67.4)	60.5 (51.2,69.8)	4.6 (−6.1,15.2)	0.413
**General combinations**									
Meeting none	165	6.5 (5.1,7.8)	4.8 (2.1,7.4)	6.2 (3.2,9.1)	5.5 (1.9,9.2)	9.3 (5.5,13.1)	6.3 (3.7,8.8)	1.5 (−2.2,5.2)	0.325
Meeting only one out of three	683	28.4 (25.0,31.8)	27.8 (22.1,33.6)	26.2 (22.4,30.0)	20.8 (15.9,25.7)	31.8 (25.2.38.5)	35.4 (29.1,41.7)	7.5 (−1.0,16.1)	0.293
Meeting any two out of three	921	40.7 (35.6,45.7)	42.5 (36.0,49.1)	45.2 (38.9,51.6)	38.0 (28.2,47.8)	38.9 (32.2,45.6)	39.1 (32.6,45.7)	−3.4 (−12.7,5.9)	0.202
Meeting all three	504	24.5 (19.8,29.2)	24.9 (19.3,30.4)	22.4 (17.0,27.9)	35.6 (21.0,50.3)	19.9 (15.8,24.0)	19.2 (14.0,24.5)	−5.7 (−13.3,2.0)	0.581
**Specific combinations**									
Only meeting PA	342	14.3 (11.9,16.7)	13.1 (8.7,17.5)	12.5 (8.5,16.6)	13.1 (7.5,18.8)	16.9 (12.5,21.3)	15.7 (9.2,22.2)	2.6 (−5.3,10.5)	0.113
Only meeting SB	124	5.6 (4.1,7.0)	8.8 (4.3,13.4)	6.2 (3.6,8.8)	3.1 (1.4,4.8)	2.8 (0.6,4.9)	7.3 (4.2,10.4)	−1.6 (−7.1,4.0)	0.522
Only meeting SLP	217	8.5 (6.7,10.3)	5.9 (1.8,9.9)	7.5 (4.6,10.3)	4.6 (2.5,6.6)	12.2 (8.5,15.9)	12.4 (8.0,16.8)	6.5 (0.6,12.5)	0.122
Meeting PA and SB	349	13.5 (11.5,15.5)	17.3 (12.5,22.2)	19.1 (14.7,23.6)	11.1 (8.6,13.5)	10.3 (5.7,14.9)	10.3 (7.0,13.5)	−7.1 (−12.9,-1.3)	0.068
Meeting PA and SLP	364	17.8 (14.1,21.5)	17.2 (10.8,23.6)	14.3 (7.8,20.8)	18.4 (10.9,26.0)	19.6 (12.6,26.6)	19.0 (11.7,26.2)	1.8 (−7.9,11.4)	0.215
Meeting SB and SLP	208	9.4 (7.3,11.5)	8.0 (5.4,10.5)	11.8 (8.0,15.6)	8.5 (5.2,11.7)	9.0 (3.2,14.7)	9.9 (5.4,14.4)	1.9 (−3.3,7.1)	0.866

Data are presented as survey-weighted prevalence (%) and 95% CI. ^*^ For adolescents aged 16–17 years: PA, an accumulation of at least 60 min/day of MVPA; SB, no more than 2 h/day of recreational screen time; SLP, uninterrupted 8 to 10 h/night of sleep. For adolescents aged 18–19 years: PA, an accumulation of at least 150 min/day of MVPA; SB, no more than 3 h/day of recreational screen time; SLP, uninterrupted 7 to 9 h/night of sleep. CI, confidence interval; NHANES, National Health and Nutrition Examination Survey; PA, physical activity; SB, sedentary behavior; SLP, sleep.

**Figure 2:**
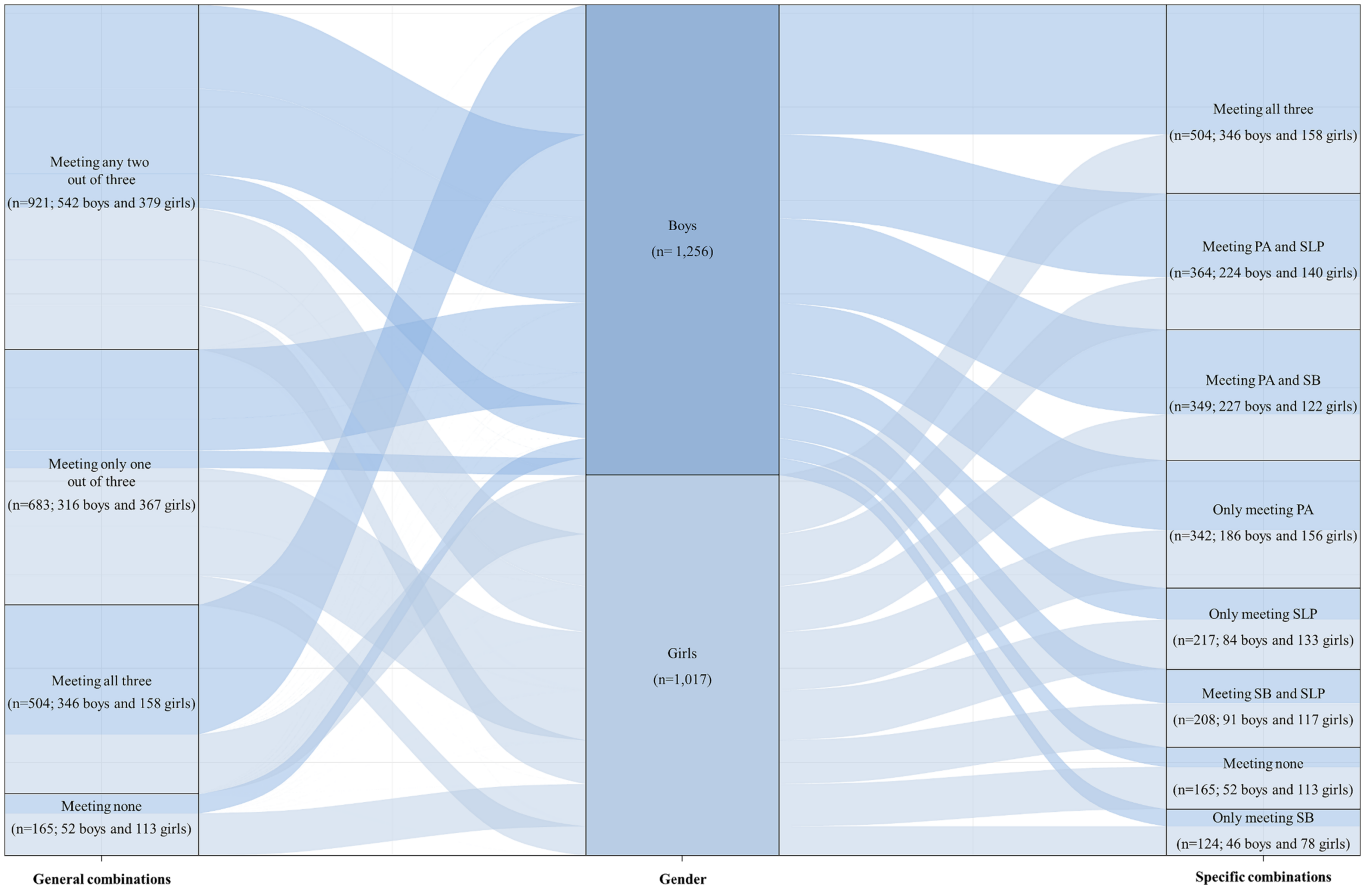
Sankey Diagram for U.S. adolescents meeting general combinations and specific combinations, stratified by gender, NHANES 2007–2016 (*n* = 2,273). Sankey Diagram is a visualization used to depict a flow from one set of values to another. The things being connected are called nodes and the connections are called links. Data flows from the gender node in the middle to general combinations node and specific combinations node, and the thickness of the links represents the amount of flowing data. General combinations include meeting none, meeting only one out of three, meeting any two out of three, and meeting all three guidelines (PA, SB, and SLP). Specific combinations include only meeting PA, only meeting SB, only meeting SLP, meeting PA and SB, meeting PA and SLP, and meeting SB and SLP. NHANES, National Health and Nutrition Examination Survey; PA, physical activity; SB, sedentary behavior; SLP, sleep.

The study found that a higher percentage of boys (29.3%, *n* = 346) met all three recommendations compared to girls (18.7%, *n* = 158). Conversely, a higher percentage of girls (10.0%, *n* = 113) did not meet any of the three recommendations (Figure 2). When considering race/ethnicity, a greater number of non-Hispanic White (*n* = 412) and Mexican American (*n* = 320) participants met the recommendation for SLP, with average SLP times of 7.70 (SE = 0.06) hours and 7.76 (SE = 0.08) hours, respectively (Supplementary Table S1). The study also revealed statistically significant differences between survey years in terms of race/ethnicity, PIR, parental education level, total energy intake, SB time, and SLP time (Supplementary Table S2).

### Trend of U.S. adolescents meeting the 24-hour movement guidelines

As shown in Table 2, there were declining patterns observed in the prevalence of meeting PA recommendation (change = −8.4%, 95% CI: −17.8% to 1.1%) or SB recommendation (change = −12.4%, 95% CI: −25.3% to 0.5%) from the 2007–2008 cycle to the 2015–2016 cycle. However, there was an increase in the prevalence of meeting SLP recommendation by 4.6% (95% CI: −6.1% to 15.2%). It was found that the prevalence (95% CI) of meeting none of the three recommendations slightly increased by 1.5% (95% CI: −2.2% to 5.2%) from the 2007–2008 cycle to the 2015–2016 cycle. Additionally, the prevalence (95% CI) of meeting all three recommendations decreased from 24.9% (19.3% to 30.4%) in the 2007–2008 cycle to 19.2% (14.0% to 24.5%) in the 2015–2016 cycle (change = −5.7%, 95% CI: −13.3% to 2.0%). Furthermore, when considering specific combinations, the prevalence (95% CI) of meeting both PA and SLP recommendations increased by 1.8% (−7.9% to 11.4%), meeting both SB and SLP recommendations increased by 1.9% (−3.3% to 7.1%), while meeting both PA and SB recommendations decreased the most by 7.1% (−12.9% to −1.3%).

**Figure 3:**
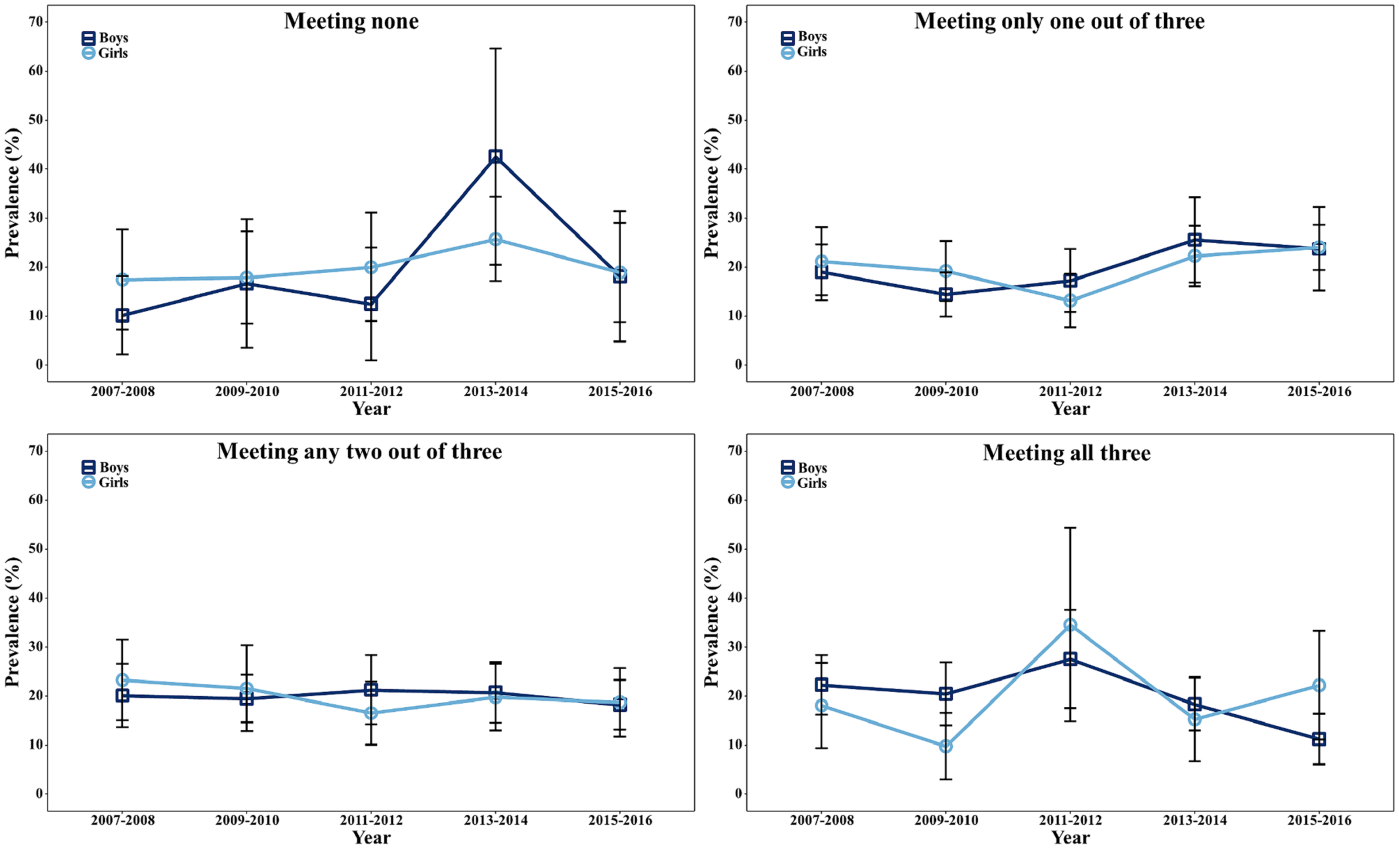
Prevalence of meeting general combinations in different survey years among U.S. adolescents aged 16–19 years, stratified by gender, NHANES 2007–2016 (*n* = 2,273). General combinations include meeting none, meeting only one out of three, meeting any two out of three, and meeting all three guidelines (PA, SB, and SLP). Prevalence in different survey years of each combination were stratified by gender (boys and girls). For example, in 2015–2016 cycle, prevalence of meeting all three guidelines for girls was higher than those in boys. NHANES, National Health and Nutrition Examination Survey; PA, physical activity; SB, sedentary behavior; SLP, sleep.

Figure 3 illustrates the secular prevalence changes of meeting general combinations of the 24-HMG recommendations, stratified by gender. It can be observed that from 2013–2014 cycle to 2015–2016 cycle, more boys met at least one recommendation compared to girls. However, during this period, more girls met all three recommendations than boys. The sharpest increase in not meeting any of the three recommendations from 2011–2012 cycle to 2013–2014 cycle was observed among boys (Supplementary Figure S1). Additionally, Supplementary Figure S2 provides an overview of the secular prevalence when stratified by race/ethnicity.

### Subgroup analysis

Table 3 and Supplementary Tables S3, S4 separately presented the results on compliance with the 24-HMG according to general combinations, individual recommendations, and specific combinations in different subgroups. For instance, with regard to general combinations, boys (29.3%, 95% CI: 24.6% to 33.9%), non-Hispanic White (25.1%, 95% CI: 19.6% to 30.6%), participants with PIR value <1.0 (28.3%, 95% CI: 21.0% to 35.7%) or appropriate total energy intake [Quartile 2 (27.8%, 95% CI: 21.2% to 34.3%)], and those whose parents attained education more than 12th grade (42.4%, 95% CI: 33.2% to 51.5%), had the highest prevalence of meeting all three recommendations (Table 3). In addition, we observed a trend effect on the numbers of recommendations met within 24-HMG among the subgroup that whose parents attained education above 12th grade (*P*_trend_ < 0.05).

**Table 3:** Subgroup analyses for prevalence of meeting the 24-hour movement guidelines among U.S. adolescents aged 16–19 years, stratified by general combinations, NHANES 2007–2016 (*n* = 2,273).

Subgroup items	Estimate U.S. population	Total participants, *n* (%)	General combinations, prevalence (95% CI)				
			Meeting none	Meeting only one out of three	Meeting any two out of three	Meeting all three	*P*_trend_
**Gender**							
Boys	36,958,988	1,256 (55.3)	3.5 (2.1,5.0)	24.7 (21.0,28.5)	42.4 (37.9,47.0)	29.3 (24.6,33.9)	0.240
Girls	30,475,600	1,017 (44.7)	10.0 (7.3,12.7)	32.8 (29.3,36.4)	38.5 (33.6,43.4)	18.7 (13.3,24.1)	0.694
**Race/ethnicity**							
Non-Hispanic White	39,661,366	690 (30.4)	5.9 (3.8,7.9)	28.0 (24.5,31.5)	41.1 (35.9,46.2)	25.1 (19.6,30.6)	0.372
Non-Hispanic Black	9,631,722	566 (24.9)	7.2 (4.6,9.8)	30.0 (25.6,34.5)	41.8 (36.8,46.8)	21.0 (15.3,26.7)	0.530
Mexican American	9,044,757	506 (22.3)	6.9 (4.2,9.7)	25.7 (20.3,31.1)	42.6 (37.4,47.8)	24.8 (19.5,30.1)	0.375
Other races (including multi-racial, other Hispanic)	9,096,743	511 (22.5)	7.7 (4.9,10.5)	31.1 (24.8,37.3)	35.7 (29.9,41.5)	25.6 (19.2,31.9)	0.389
**PIR**							
Below poverty (<1.0)	15,907,136	707 (31.1)	5.7 (3.8,7.6)	23.9 (19.8,28.0)	42.1 (35.3,48.8)	28.3 (21.0,35.7)	0.261
Above poverty (≥1.0)	51,527,451	1,566 (68.9)	6.7 (5.0,8.4)	29.8 (26.8,32.7)	40.2 (36.2,44.2)	23.3 (18.8,27.9)	0.447
**Parental education level**							
Less than 9th grade	1,901,955	71 (3.1)	12.4 (−3.0,27.9)	21.1 (8.3,34.0)	48.5 (30.9,66.1)	17.9 (7.2,28.6)	0.648
9–12th grade (including 12th grade with no diploma)	55,344,165	1,897 (83.5)	6.9 (5.4,8.4)	30.6 (27.7,33.6)	41.0 (37.4,44.6)	21.4 (18.1,24.8)	0.519
More than 12th grade	10,188,467	305 (13.4)	2.8 (1.0, 4.5)	17.6 (11.8,23.3)	37.3 (28.5,46.1)	42.4 (33.2,51.5)	**<0.05**
**Total energy intake, kcal**							
Quartile 1 (76, 1505]	16,626,939	563 (24.8)	8.9 (5.3,12.5)	28.3 (23.1,33.6)	42.4 (36.5,48.4)	20.3 (15.0,25.6)	0.557
Quartile 2 (1505, 2014]	15,872,385	568 (25.0)	6.9 (3.7,10.1)	32.9 (26.9,38.9)	32.5 (26.5,38.4)	27.8 (21.2,34.3)	0.346
Quartile 3 (2014, 2692]	17,927,290	576 (25.3)	5.4 (3.6,7.3)	29.0 (24.4,33.5)	40.6 (33.3,48.0)	25.0 (17.6,32.4)	0.379
Quartile 4 (2692, 10436)	17,007,973	566 (24.9)	4.7 (2.9,6.5)	23.7 (18.1,29.2)	46.6 (41.1,52.1)	25.0 (20.5,29.6)	0.368
**Survey years**							
2007–2008	13,862,963	429 (18.9)	4.8 (2.1,7.5)	27.8 (22.1,33.6)	42.5 (36.0,49.1)	24.9 (19.3,30.4)	0.376
2009–2010	12,375,849	490 (21.6)	6.2 (3.2,9.1)	26.2 (22.4,30.0)	45.2 (38.9,51.6)	22.4 (17.0,27.9)	0.455
2011–2012	13,911,006	423 (18.6)	5.6 (1.9,9.2)	20.8 (15.9,25.8)	38.0 (28.2,47.8)	35.6 (21.0,50.3)	0.078
2013–2014	14,347,859	497 (21.9)	9.3 (5.5,13.2)	31.9 (25.2,38.5)	38.9 (32.2,45.6)	19.9 (15.8,24.0)	0.617
2015–2016	12,936,911	434 (19.1)	6.3 (3.7,8.8)	35.4 (29.1,41.7)	39.1 (32.6,45.7)	19.2 (14.0,24.5)	0.639

Data are presented as survey-weighted prevalence (%) and 95% CI. *P*_trend_ presented with bold valued were statistically significant. CI, confidence interval; NHANES, National Health and Nutrition Examination Survey; PIR, poverty to income ratio.

## Discussion

Based on a nationally representative sample of U.S. adolescents in NHANES 2007–2016, this study provides an important update on the prevalence and trends of U.S. adolescents meeting the 24-HMG. Overall, a relatively limited proportion (< 30%) of U.S. adolescents reported meeting all three recommendations simultaneously for PA, SB, and SLP, and this pattern remained consistent from 2007–2008 cycle to 2015–2016 cycle. Although more adolescents achieved the SLP guidelines (increased by 4.6%), there was a lower prevalence of meeting the recommendations for PA (decreased by 8.4%) and SB (decreased by 12.4%). Additionally, there were noticeable differences in the number of meeting the three recommendations based on parental education level.

Our findings are in line with previous studies, which have shown that around 80% of adolescents worldwide are not getting enough PA, either due to reduced PA or increased SB (31). The updated 2nd edition of the Physical Activity Guidelines for Americans (32) provides evidence-based recommendations for youth aged 6–17, stating that they should engage in at least 60 min of MVPA per day, including aerobic activities as well as those that strengthen muscles and bones. In our study, we observed that 70.1% of U.S. adolescents aged 16–19 met PA guidelines within a 24-hour measurement period from 2007 to 2016. However, there has been a concerning decline in recent years, which is consistent with the global trend that the majority of adolescents not meeting the current PA recommendations (33). Consistent largely with previous studies (21, 34), we found that although a high proportion of adolescents achieved the recommended amount of SLP, there was a low level and a decrease (change = −12.4%) in the number of U.S. adolescents meeting the SB guidelines. It has been documented that the increase in SB is related to many social and lifestyle factors, one of which is Internet gaming disorder (IGD) resulting from rapidly evolving technology (35). Currently, the prevalence of IGD in adolescents in North America is 9.4% (36). Given the worrying status of internet gaming disorder (IGD) and the link between SB and IGD, high levels of SB may not only exist but also persist. Additionally, in recent years, there have been concerning changes in the 24-hour movement behaviors of adolescents during the COVID-19 pandemic, such as increases in recreational screen time and significant decreases in PA. Meanwhile, these behaviors interact with each other, which means that a rise in SB is often accompanied by a decline in PA (37). It has been demonstrated that adherence to 24-HMG was associated with several benefits, such as better academic achievement among adolescents (38) and better health indicators in adulthood (12). Considering these potential benefits that result from daily movement behaviors and our alarming findings, the state of the 24-HMG among U.S. adolescents is troubling.

Another key finding of this study is the decrease observed from 2007 to 2016 in the number of individuals who were able to meet all three recommendations within the 24-HMG. Previous studies have also reported similar findings, indicating that only a small percentage of U.S. high school students (3% of girls; 7% of boys) are able to achieve the optimal amount of SLP, PA, and limited screen time (39). Compared to other studies, this research utilized data from NHANES to identify a higher prevalence (24.5%) of U.S. adolescents meeting all three recommendations. This difference can be attributed to the variation in the age range of the study population. In this study, the participants were adolescents aged 16–19 years, whereas other studies included participants aged below 17 years (21), all ages (39), or aged 9–11 years (40). Previous studies have emphasized the importance of considering covariates such as socio-demographic factors (e.g., age, gender, race), parenting style, and home environment when examining the disparities in meeting guidelines (21, 39). This study also found that a higher percentage of boys (29.3%) met all three recommendations compared to girls (18.7%), while a study conducted in China shown no statistically significant percentage difference between girls and boys in meeting 24-HMG guidelines, independently or jointly (41). This inconsistency may be due to differences in race and culture, so further research is necessary to identify the effect of gender difference on adherence to 24-HMG. Additionally, our research suggests that parental education may significantly influence the likelihood of meeting all three guidelines. This may be due to the fact that parental education is one of the most important factors of socioeconomic status that proved to be an indispensable role in the formation of familial social background and children’s behavioral patterns (42). Meanwhile, research has found the higher the education level of parents, the longer they spend with their children, which may help their children develop healthy habits (43). Therefore, policy makers and clinicians should prioritize identifying and intervening with socio-demographic groups that are less likely to adhere to the 24-HMG, in order to mitigate the negative impacts of low adherence (44).

This study had several notable strengths. First, it was based on a large sample of U.S. adolescents who participated in NHANES, allowing for a comprehensive characterization of the prevalence of adherence to 24-HMG. Second, the study employed ten consecutive years of data to evaluate the trend over this period, providing an overview of the prevalence meeting 24-HMG. This study has several limitations that should be mentioned. First, the cross-sectional design of the study prevents us from drawing causal inferences about the prevalence of U.S. adolescents meeting the 24-HMG, and has limitations in capturing long-term trends. Second, there may be some bias in using the Canadian 24-HMG to assess the prevalence among the U.S. population. Third, the self-reported data for the three movement behaviors may be subject to information bias, such as recall bias. Additionally, while the measures for SB and SLP were assessed on a daily basis, PA data were only available on a weekly basis. This makes it difficult to account for weekday-weekend variations in PA. Future studies should focus on addressing these limitations by estimating movement patterns across the 24-hour period, taking into account differences between weekdays and weekends. Meanwhile, further analysis methods, such as network analysis, can be used to more clearly quantify the importance of each behavior. This will provide a more comprehensive understanding of the prevalence discrepancy and help inform public health interventions and guidelines for improving the overall health of adolescents.

## Conclusion

The estimated prevalence of meeting the recommendations within 24-HMG among the U.S. adolescent population remained relatively stable at a low level from 2007 to 2016. However, there are disparities in the prevalence of meeting all three recommendations based on parental education levels. Further research is necessary to identify the factors contributing to the low level of adherence to 24-HMG among U.S. adolescents.

## Data availability statement

The original contributions presented in the study are included in the article/Supplementary material, further inquiries can be directed to the corresponding author.

## Ethics statement

The research ethics review boards of National Center for Health Statistics authorized the NHANES study protocols and all participants provided written informed consent.

## Author contributions

X-qL: Data curation, Methodology, Resources, Validation, Writing – original draft. M-lL: Data curation, Investigation, Visualization, Writing – original draft. Z-wW: Resources, Data curation, Writing – review & editing. J-hL: Data curation, Methodology, Project administration, Resources, Software, Supervision, Validation, Writing – review & editing.
