# Supplementary material for: Secular trends in the prevalence of meeting 24-hour movement guidelines among U.S. adolescents: evidence from NHANES 2007–2016
Source: Front Public Health. 2024 Apr 3;12:1362718. doi: 10.3389/fpubh.2024.1362718 (PMC11021726; doi:10.3389/fpubh.2024.1362718)
Supplement: Supplementary file 1 [file Data_Sheet_1.docx]

**Supplementary Materials**

**Secular Trends in the Prevalence of Meeting 24-Hour Movement Guidelines among U.S. Adolescents: Evidence from NHANES 2007-2016**

**List of Contents**

[Supplementary Figure S1. Proportions of U.S. adolescents in different survey years meeting 24-Hour Movement Guidelines, stratified by gender, NHANES 2007-2016 (n= 2,273). 3](#_Toc161481582)

[Supplementary Figure S2. Proportions of U.S. adolescents in different survey years meeting 24-Hour Movement Guidelines, stratified by race/ethnicity, NHANES 2007-2016 (n= 2,273). 4](#_Toc161481583)

[Supplementary Table S1. Demographic characteristics variables of U.S. adolescents aged 16-19 years stratified by race/ethnicity, NHANES 2007-2016 (n= 2,273). 5](#_Toc161481584)

[Supplementary Table S2. Demographic characteristics variables of U.S. adolescents aged 16-19 years stratified by survey years, NHANES 2007-2016 (n= 2,273). 6](#_Toc161481585)

[Supplementary Table S3. Subgroup analyses for prevalence of meeting 24-Hour Movement Guidelines among U.S. adolescents aged 16-19 years, stratified by individual recommendations, NHANES 2007-2016 (n= 2,273). 7](#_Toc161481586)

[Supplementary Table S4. Subgroup analyses for prevalence of meeting 24-Hour Movement Guidelines among U.S. adolescents aged 16-19 years, stratified by specific combinations, NHANES 2007-2016 (n= 2,273). 8](#_Toc161481587)


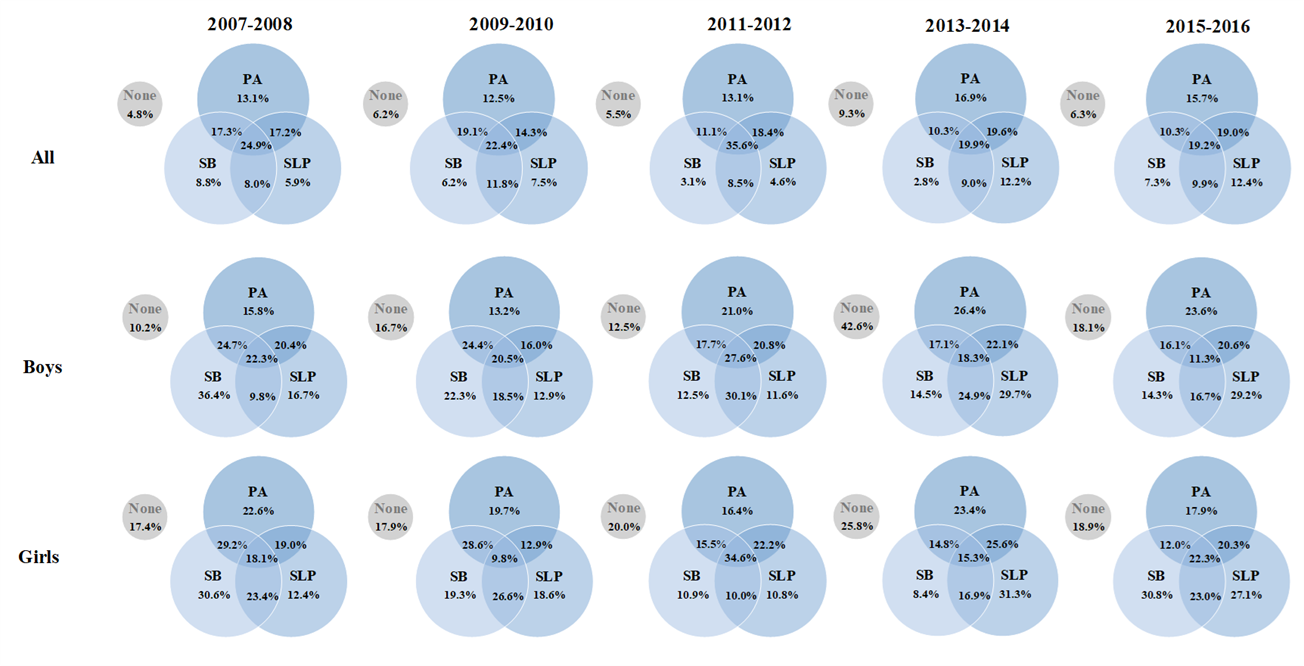


# Supplementary Figure S1. Proportions of U.S. adolescents in different survey years meeting 24-Hour Movement Guidelines, stratified by gender, NHANES 2007-2016 (n= 2,273).

Abbreviations: NHANES, National Health and Nutrition Examination Survey; PA, physical activity; SB, sedentary behavior; SLP, sleep.


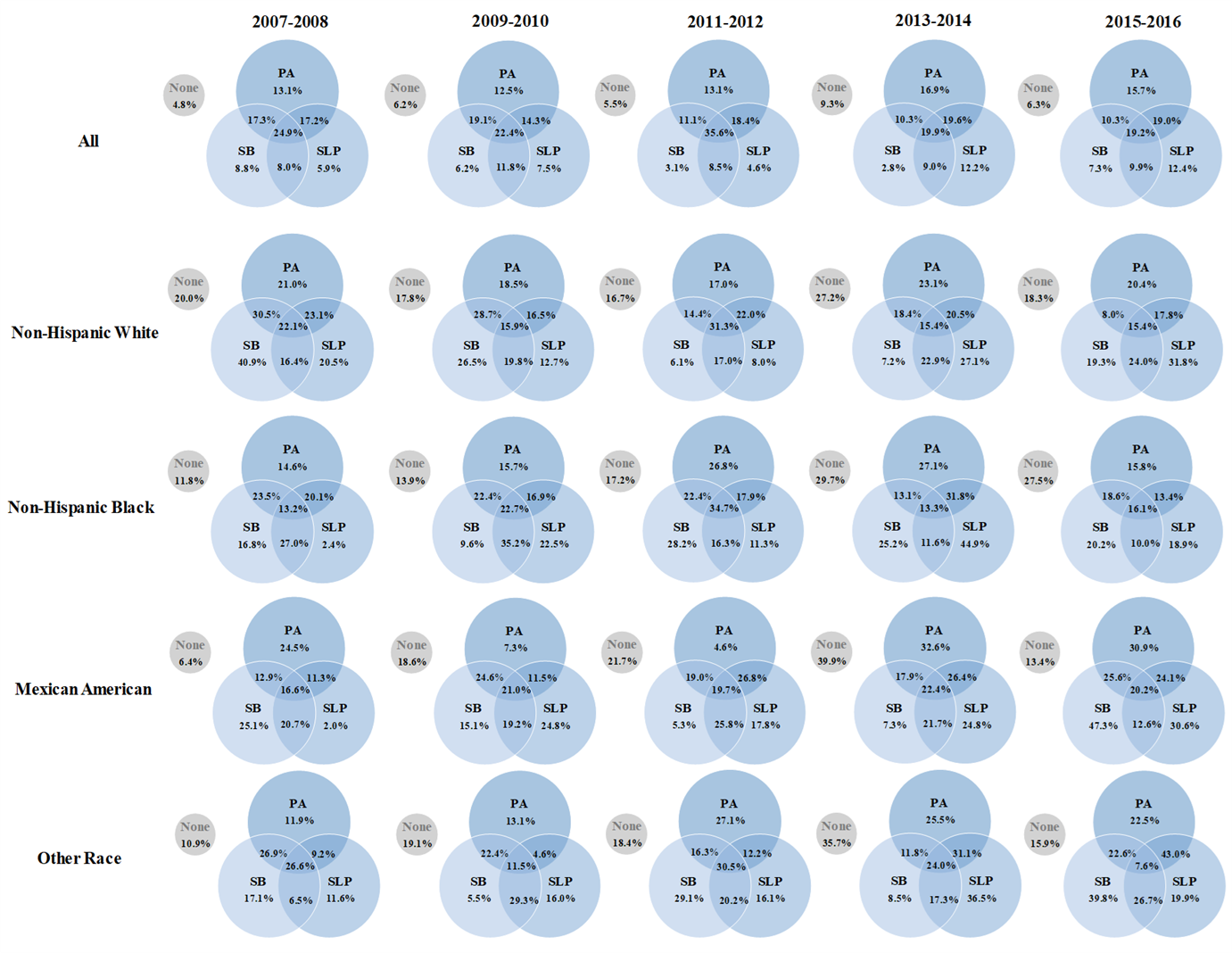


# Supplementary Figure S2. Proportions of U.S. adolescents in different survey years meeting 24-Hour Movement Guidelines, stratified by race/ethnicity, NHANES 2007-2016 (n= 2,273).

Abbreviations: MVPA, moderate to vigorous physical activity; NHANES, National Health and Nutrition Examination Survey; SB, sedentary behavior; SLP, sleep.

| Supplementary Table S1. Demographic characteristics variables of U.S. adolescents aged 16-19 years stratified by race/ethnicity, NHANES 2007-2016 (n= 2,273). | | | | | | | |
| --- | --- | --- | --- | --- | --- | --- | --- |
| **Characteristics** | **Estimate U.S. population** | **Total participants** | **Race/ethnicity** | | | | ***p*-value** |
|  |  |  | **Non-Hispanic White** | **Non-Hispanic Black** | **Mexican American** | **Other races** |  |
| **No. of participants** | 67,434,588 | 2,273 (100.0) | 690 (30.4) | 566 (24.9) | 506 (22.3) | 511 (22.5) | **-** |
| **Age, years** | - | 17.44 ± 0.05 | 17.41 ± 0.06 | 17.50 ± 0.10 | 17.44 ± 0.07 | 17.47 ± 0.08 | 0.72 |
| **Gender** |  |  |  |  |  |  |  |
| Boys | 36,958,988 | 1,256 (55.3) | 388 (54.5) | 318 (53.2) | 283 (58.3) | 267 (54.6) | 0.60 |
| Girls | 30,475,600 | 1,017 (44.7) | 302 (45.5) | 248 (46.8) | 223 (41.7) | 244 (45.4) |  |
| **BMI, kg/m^2^** | - | 25.03 ± 0.18 | 24.45 ± 0.26 | 26.46 ± 0.36 | 26.18 ± 0.39 | 24.93 ± 0.44 | **< 0.001** |
| **PIR** | - | 2.45 ± 0.09 | 2.88 ± 0.11 | 1.74 ± 0.13 | 1.60 ± 0.08 | 2.13 ± 0.12 | **< 0.001** |
| Below poverty (< 1.0) | 15,907,136 | 707 (31.1) | 143 (15.4) | 215 (39.9) | 184 (37.7) | 165 (27.9) | **< 0.001** |
| Above poverty (≥ 1.0) | 51,527,451 | 1,566 (68.9) | 547 (84.6) | 351 (60.1) | 322 (62.3) | 346 (72.1) |  |
| **Parental education level** |  |  |  |  |  |  |  |
| Less than 9th grade | 1,901,955 | 71 (3.1) | 23 (2.7) | 17 (2.6) | 16 (3.7) | 15 (2.9) | 0.36 |
| 9-12th grade (including 12th grade with no diploma) | 55,344,165 | 1,897 (83.5) | 577 (82.0) | 454 (78.0) | 436 (85.2) | 430 (83.7) |  |
| More than 12th grade | 10,188,467 | 305 (13.4) | 90 (15.4) | 95 (19.4) | 54 (11.0) | 66 (13.5) |  |
| **Total energy intake, kcal** | - | 2235.18 ± 29.39 | 2287.37 ± 44.90 | 2177.58 ± 42.57 | 2154.60 ± 49.82 | 2148.72 ± 73.35 | 0.15 |
| Quartile 1 (76,1505] | 16,626,939 | 563 (24.8) | 156 (22.6) | 150 (26.5) | 120 (23.7) | 137 (26.8) | 0.80 |
| Quartile 2 (1505,2014] | 15,872,385 | 568 (25.0) | 172 (24.9) | 133 (23.5) | 134 (26.5) | 129 (25.2) |  |
| Quartile 3 (2014,2692] | 17,927,290 | 576 (25.3) | 182 (26.4) | 143 (25.3) | 126 (24.9) | 125 (24.5) |  |
| Quartile 4 (2692,10436) | 17,007,973 | 566 (24.9) | 180 (26.1) | 140 (24.7) | 126 (24.9) | 120 (23.5) |  |
| **MVPA, min/week** | - | 1280.30 ± 40.03 | 1298.25 ± 58.92 | 1295.95 ± 77.41 | 1150.63 ± 78.21 | 1314.39 ± 108.08 | 0.41 |
| **SB, min/day** | - | 414.57 ± 6.98 | 414.64 ± 9.73 | 414.25 ± 10.39 | 402.57 ± 14.03 | 426.54 ± 11.49 | 0.66 |
| **SLP time, h/night** | - | 7.61 ± 0.04 | 7.70 ± 0.06 | 7.29 ± 0.08 | 7.76 ± 0.08 | 7.44 ± 0.08 | **< 0.001** |
| **Individual recommendations^*^** |  |  |  |  |  |  |  |
| Meeting PA | 47,283,726 | 1,559 (68.6) | 487 (71.2) | 390 (68.7) | 346 (66.6) | 336 (70.4) | 0.58 |
| Meeting SB | 35,709,776 | 1,185 (52.1) | 346 (51.7) | 318 (56.9) | 276 (55.0) | 245 (52.0) | 0.54 |
| Meeting SLP | 40,553,337 | 1,293 (56.9) | 412 (62.3) | 282 (51.0) | 320 (63.6) | 279 (56.7) | **< 0.05** |
| **General combinations** |  |  |  |  |  |  |  |
| Meeting none | 4,349,612 | 165 (7.3) | 51 (5.9) | 37 (7.2) | 33 (6.9) | 44 (7.7) | 0.71 |
| Meeting only one out of three | 19,144,891 | 683 (30.1) | 194 (28.0) | 179 (30.0) | 133 (25.7) | 177 (31.1) |  |
| Meeting any two out of three | 27,418,306 | 921 (40.5) | 284 (41.1) | 239 (41.8) | 211 (42.6) | 187 (35.7) |  |
| Meeting all three | 16,521,779 | 504 (22.2) | 161 (25.1) | 111 (21.0) | 129 (24.8) | 103 (25.6) |  |
| **Specific combinations** |  |  |  |  |  |  |  |
| Only meeting PA | 9,654,649 | 342 (15.1) | 98 (14.1) | 96 (15.8) | 60 (10.7) | 88 (17.3) | 0.07 |
| Only meeting SB | 3,760,613 | 124 (5.5) | 35 (5.7) | 41 (7.1) | 29 (6.3) | 19 (2.7) |  |
| Only meeting SLP | 5,729,629 | 217 (9.6) | 61 (8.2) | 42 (7.2) | 44 (8.7) | 70 (11.1) |  |
| Meeting PA and SB | 9,116,376 | 349 (15.4) | 94 (12.0) | 110 (18.9) | 64 (12.4) | 81 (15.6) |  |
| Meeting PA and SLP | 11,990,922 | 364 (16.0) | 134 (20.1) | 73 (13.0) | 93 (18.7) | 64 (11.9) |  |
| Meeting SB and SLP | 6,311,007 | 208 (9.2) | 56 (9.0) | 56 (9.9) | 54 (11.5) | 42 (8.1) |  |
| **Survey years** |  |  |  |  |  |  |  |
| 2007-2008 | 13,862,963 | 429 (18.9) | 154 (22.3) | 110 (19.4) | 96 (19.0) | 69 (13.5) | **< 0.001** |
| 2009-2010 | 12,375,849 | 490 (21.6) | 170 (24.6) | 114 (20.1) | 132 (26.1) | 74 (14.5) |  |
| 2011-2012 | 13,911,006 | 423 (18.6) | 100 (14.5) | 140 (24.7) | 66 (13.0) | 117 (22.9) |  |
| 2013-2014 | 14,347,859 | 497 (21.9) | 134 (19.4) | 114 (20.1) | 116 (22.9) | 133 (26.0) |  |
| 2015-2016 | 12,936,911 | 434 (19.1) | 132 (19.1) | 88 (15.5) | 96 (19.0) | 118 (23.1) |  |
| Footnotes: Data are presented as counts (n) and percentage (%) for categorical variables or mean ± SE for numerical variables. *P*-values were calculated using t-test or Mann-Whitney U test (numerical variables) or using chi-square test or Fisher exact test (categorical variables) to represent the differences between boys and girls. *P*-values presented with bold valued were statistically significant. ^*^ For adolescents aged 16-17 years: PA, an accumulation of at least 60 min/day of MVPA; SB, no more than 2 h/day of recreational screen time; SLP, uninterrupted 8 to 10 h/night of sleep. For adolescents aged 18-19 years: PA, an accumulation of at least 150 min/day of MVPA; SB, no more than 3 h/day of recreational screen time; SLP, uninterrupted 7 to 9 h/night of sleep. BMI, body mass index; MVPA, moderate to vigorous physical activity; NHANES, National Health and Nutrition Examination Survey; PA, physical activity; PIR, poverty to income ratio; SB, sedentary behavior; SE, standard error; SLP, sleep. | | | | | | | |

| Supplementary Table S2. Demographic characteristics variables of U.S. adolescents aged 16-19 years stratified by survey years, NHANES 2007-2016 (n= 2,273). | | | | | | | | |
| --- | --- | --- | --- | --- | --- | --- | --- | --- |
| **Characteristics** | **Estimate U.S. population** | **Total participants** | **Survey years** | | | | | ***p*-value** |
|  |  |  | **2007-2008** | **2009-2010** | **2011-2012** | **2013-2014** | **2015-2016** |  |
| **No. of participants** | 67,434,588 | 2,273 (100.0) | 429 (18.9) | 490 (21.6) | 423 (18.6) | 497 (21.9) | 434 (19.1) | - |
| **Age, years** | - | 17.44 ± 0.05 | 17.42 ± 0.09 | 17.38 ± 0.08 | 17.65 ± 0.16 | 17.42 ± 0.07 | 17.29 ± 0.04 | 0.14 |
| **Gender** |  |  |  |  |  |  |  |  |
| Boys | 36,958,988 | 1,256 (55.3) | 243 (53.7) | 294 (55.1) | 233 (58.0) | 261 (56.7) | 225 (50.2) | 0.60 |
| Girls | 30,475,600 | 1,017 (44.7) | 186 (46.3) | 196 (44.9) | 190 (42.0) | 236 (43.3) | 209 (49.8) |  |
| **Race/ethnicity** |  |  |  |  |  |  |  |  |
| Non-Hispanic White | 39,661,366 | 690 (30.4) | 154 (35.9) | 170 (34.7) | 100 (23.6) | 134 (27.0) | 132 (30.4) | **< 0.001** |
| Non-Hispanic Black | 9,631,722 | 566 (25.0) | 110 (25.6) | 114 (23.3) | 140 (33.1) | 114 (22.9) | 88 (20.3) |  |
| Mexican American | 9,044,757 | 506 (22.3) | 96 (22.4) | 132 (26.9) | 66 (15.6) | 116 (23.3) | 96 (22.1) |  |
| Other races (including multi-racial, other Hispanic) | 9,096,743 | 511 (22.5) | 69 (16.1) | 74 (15.1) | 117 (27.7) | 133 (26.8) | 118 (27.2) |  |
| **BMI, kg/m^2^** | - | 25.03 ± 0.18 | 24.53 ± 0.43 | 25.32 ± 0.31 | 24.29 ± 0.32 | 25.68 ± 0.50 | 25.37 ± 0.40 | 0.06 |
| **PIR** | - | 2.45 ± 0.09 | 2.67 ± 0.19 | 2.60 ± 0.14 | 2.10 ± 0.30 | 2.30 ± 0.13 | 2.58 ± 0.18 | 0.25 |
| Below poverty (< 1.0) | 15,907,136 | 707 (31.1) | 125 (29.1) | 142 (29.0) | 151 (35.7) | 173 (34.8) | 116 (26.7) | **< 0.05** |
| Above poverty (≥ 1.0) | 51,527,451 | 1,566 (68.9) | 304 (70.9) | 348 (71.0) | 272 (64.3) | 324 (65.2) | 318 (73.3) |  |
| **Parental education level** |  |  |  |  |  |  |  |  |
| Less than 9th grade | 1,901,955 | 71 (3.1) | 20 (3.9) | 13 (1.7) | 14 (4.0) | 12 (1.5) | 12 (3.0) | **< 0.05** |
| 9-12th grade (including 12th grade with no diploma) | 55,344,165 | 1,897 (83.5) | 346 (80.9) | 411 (83.8) | 325 (70.1) | 432 (87.9) | 383 (88.1) |  |
| More than 12th grade | 10,188,467 | 305 (13.4) | 63 (15.2) | 66 (14.5) | 84 (25.9) | 53 (10.6) | 39 (9.0) |  |
| **Total energy intake, kcal** | - | 2235.18 ± 29.39 | 2291.19 ± 55.73 | 2241.18 ± 61.20 | 2390.35 ± 69.03 | 2173.15 ± 43.17 | 2071.35 ± 87.46 | **< 0.05** |
| Quartile 1 (76,1505] | 16,626,939 | 563 (24.8) | 99 (23.1) | 99 (20.2) | 108 (25.5) | 133 (26.8) | 124 (28.6) | **< 0.05** |
| Quartile 2 (1505,2014] | 15,872,385 | 568 (25.0) | 106 (24.7) | 124 (25.3) | 95 (22.5) | 130 (26.2) | 113 (26.0) |  |
| Quartile 3 (2014,2692] | 17,927,290 | 576 (25.3) | 98 (22.8) | 132 (26.9) | 118 (27.9) | 120 (24.1) | 108 (24.9) |  |
| Quartile 4 (2692,10436) | 17,007,973 | 566 (24.9) | 126 (29.4) | 135 (27.6) | 102 (24.1) | 114 (22.9) | 89 (20.5) |  |
| **MVPA, min/week** | - | 1280.30 ± 40.03 | 1356.47 ±121.15 | 1171.61 ± 70.94 | 1351.27 ± 79.11 | 1251.89 ± 89.37 | 1257.86 ± 76.57 | 0.48 |
| **SB, min/day** | - | 414.57 ± 6.98 | 389.60 ± 12.75 | 390.91 ± 15.64 | 386.15 ± 16.18 | 450.53 ± 16.38 | 454.64 ± 12.92 | **< 0.001** |
| **SLP time, h/night** | - | 7.61 ± 0.04 | 7.42 ± 0.10 | 7.40 ± 0.10 | 7.63 ± 0.06 | 7.45 ± 0.09 | 8.19 ± 0.07 | **< 0.001** |
| **Individual recommendations^*^** |  |  |  |  |  |  |  |  |
| Meeting PA | 47,283,726 | 1,559 (68.6) | 316 (72.5) | 325 (68.4) | 298 (78.3) | 332 (66.7) | 288 (64.2) | **< 0.05** |
| Meeting SB | 35,709,776 | 1,185 (52.1) | 261 (59.0) | 300 (59.5) | 236 (58.3) | 185 (41.9) | 203 (46.6) | **< 0.05** |
| Meeting SLP | 40,553,337 | 1,293 (56.9) | 235 (55.9) | 290 (56.0) | 237 (67.1) | 283 (60.7) | 248 (60.5) | 0.19 |
| **General combinations** |  |  |  |  |  |  |  |  |
| Meeting none | 4,349,612 | 165 (7.3) | 20 (4.7) | 31 (6.3) | 29 (6.9) | 51 (10.3) | 34 (7.8) | **< 0.001** |
| Meeting only one out of three | 19,144,891 | 683 (30.1) | 118 (27.5) | 124 (25.3) | 122 (28.8) | 179 (36.0) | 140 (32.3) |  |
| Meeting any two out of three | 27,418,306 | 921 (40.5) | 179 (41.7) | 214 (43.7) | 167 (39.5) | 180 (36.2) | 181 (41.7) |  |
| Meeting all three | 16,521,779 | 504 (22.2) | 112 (26.1) | 121 (24.7) | 105 (24.8) | 87 (17.5) | 79 (18.2) |  |
| **Specific combinations** |  |  |  |  |  |  |  |  |
| Only meeting PA | 9,654,649 | 342 (15.1) | 65 (15.2) | 51 (10.4) | 66 (15.6) | 97 (19.5) | 63 (14.5) | **< 0.001** |
| Only meeting SB | 3,760,613 | 124 (5.5) | 29 (6.8) | 27 (5.5) | 26 (6.2) | 14 (2.8) | 28 (6.5) |  |
| Only meeting SLP | 5,729,629 | 217 (9.6) | 24 (5.6) | 46 (9.4) | 30 (7.1) | 68 (13.7) | 49 (11.3) |  |
| Meeting PA and SB | 9,116,376 | 349 (15.4) | 80 (18.6) | 91 (18.6) | 65 (15.4) | 52 (10.5) | 61 (14.1) |  |
| Meeting PA and SLP | 11,990,922 | 364 (16.0) | 59 (13.8) | 62 (12.7) | 62 (14.7) | 96 (19.3) | 85 (19.6) |  |
| Meeting SB and SLP | 6,311,007 | 208 (9.2) | 40 (9.3) | 61 (12.4) | 40 (9.5) | 32 (6.4) | 35 (8.1) |  |
| Footnotes: Data are presented as counts (n) and percentage (%) for categorical variables or mean ± SE for numerical variables. *P*-values were calculated using t-test or Mann-Whitney U test (numerical variables) or using chi-square test or Fisher exact test (categorical variables) to represent the differences between boys and girls. *P*-values presented with bold valued were statistically significant. ^*^ For adolescents aged 16-17 years: PA, an accumulation of at least 60 min/day of MVPA; SB, no more than 2 h/day of recreational screen time; SLP, uninterrupted 8 to 10 h/night of sleep. For adolescents aged 18-19 years: PA, an accumulation of at least 150 min/day of MVPA; SB, no more than 3 h/day of recreational screen time; SLP, uninterrupted 7 to 9 h/night of sleep. BMI, body mass index; MVPA, moderate to vigorous physical activity; NHANES, National Health and Nutrition Examination Survey; PA, physical activity; PIR, poverty to income ratio; SB, sedentary behavior; SE, standard error; SLP, sleep. | | | | | | | | |

| Supplementary Table S3. Subgroup analyses for prevalence of meeting 24-Hour Movement Guidelines among U.S. adolescents aged 16-19 years, stratified by individual recommendations, NHANES 2007-2016 (n= 2,273). | | | | | |
| --- | --- | --- | --- | --- | --- |
| **Subgroup items** | **Estimate U.S. population** | **Total participants, *n* (%)** | **Individual recommendations^*^, prevalence (95% CI)** | | |
|  |  |  | **Meeting PA** | **Meeting SB** | **Meeting SLP** |
| **Gender** |  |  |  |  |  |
| Boys | 36,958,988 | 1,256 (55.3) | 78.8 (75.4,82.3) | 55.8 (50.9,60.7) | 62.9 (58.4,67.4) |
| Girls | 30,475,600 | 1,017 (44.7) | 59.6 (54.3,64.9) | 49.5 (43.0,56.1) | 56.8 (52.1,61.5) |
| **Race/ethnicity** |  |  |  |  |  |
| Non-Hispanic White | 39,661,366 | 690 (30.4) | 71.2 (66.4,76.0) | 51.8 (45.5,58.1) | 62.3 (57.8,66.9) |
| Non-Hispanic Black | 9,631,722 | 566 (24.9) | 68.7 (63.6,73.8) | 56.9 (51.1,62.6) | 51.0 (45.4,56.6) |
| Mexican American | 9,044,757 | 506 (22.3) | 66.6 (60.7,72.5) | 55.0 (47.6,62.4) | 63.6 (57.7,69.6) |
| Other races (including multi-racial, other Hispanic) | 9,096,743 | 511 (22.5) | 70.4 (65.1,75.8) | 52.0 (46.1,57.8) | 56.7 (50.4,63.0) |
| **PIR** |  |  |  |  |  |
| Below poverty (< 1.0) | 15,907,136 | 707 (31.1) | 71.4 (66.9,75.9) | 60.2 (53.4,67.0) | 61.4 (56.5,66.4) |
| Above poverty (≥ 1.0) | 51,527,451 | 1,566 (68.9) | 69.7 (65.9,73.6) | 50.7 (45.5,55.9) | 59.7 (56.0,63.5) |
| **Parental education level** |  |  |  |  |  |
| Less than 9th grade | 1,901,955 | 71 (3.1) | 60.1 (43.4,76.9) | 53.5 (36.3,70.6) | 58.3 (40.9,75.7) |
| 9-12th grade (including 12th grade with no diploma) | 55,344,165 | 1,897 (83.5) | 69.6 (66.6,72.7) | 49.8 (45.5,54.1) | 57.5 (53.9,61.1) |
| More than 12th grade | 10,188,467 | 305 (13.4) | 74.6 (67.2,82.1) | 70.0 (62.9,77.1) | 74.7 (67.5,81.8) |
| **Total energy intake, kcal** |  |  |  |  |  |
| Quartile 1 (76,1505] | 16,626,939 | 563 (24.8) | 62.9 (55.7,70.2) | 55.0 (48.9,61.2) | 56.2 (49.4,63.0) |
| Quartile 2 (1505,2014] | 15,872,385 | 568 (25.0) | 71.2 (65.6,76.7) | 50.5 (43.5,57.6) | 59.4 (53.8,65.0) |
| Quartile 3 (2014,2692] | 17,927,290 | 576 (25.3) | 70.2 (64.7,75.7) | 49.0 (42.0,56.0) | 65.9 (60.7,71.2) |
| Quartile 4 (2692,10436) | 17,007,973 | 566 (24.9) | 76.1 (70.7,81.4) | 57.4 (51.0,63.8) | 58.6 (52.3,64.8) |
| **Survey years** |  |  |  |  |  |
| 2007-2008 | 13,862,963 | 429 (18.9) | 72.5 (65.9,79.2) | 59.0 (49.6,68.4) | 55.9 (50.7,61.1) |
| 2009-2010 | 12,375,849 | 490 (21.6) | 68.4 (64.2,72.6) | 59.5 (51.4,67.6) | 56.0 (49.9,62.1) |
| 2011-2012 | 13,911,006 | 423 (18.6) | 78.3 (70.4,86.1) | 58.3 (46.4,70.2) | 67.2 (59.2,75.1) |
| 2013-2014 | 14,347,859 | 497 (21.9) | 66.7 (60.2,73.3) | 41.9 (32.3,51.6) | 60.7 (54.0,67.4) |
| 2015-2016 | 12,936,911 | 434 (19.1) | 64.2 (57.4,70.9) | 46.6 (37.8,55.5) | 60.5 (51.2,69.8) |
| Footnotes: Data are presented as survey-weighted prevalence (%) and 95% CI. ^*^ For adolescents aged 16-17 years: PA, an accumulation of at least 60 min/day of MVPA; SB, no more than 2 h/day of recreational screen time; SLP, uninterrupted 8 to 10 h/night of sleep. For adolescents aged 18-19 years: PA, an accumulation of at least 150 min/day of MVPA; SB, no more than 3 h/day of recreational screen time; SLP, uninterrupted 7 to 9 h/night of sleep. CI, confidence interval; NHANES, National Health and Nutrition Examination Survey; PA, physical activity; PIR, poverty to income ratio; SB, sedentary behavior; SLP, sleep. | | | | | |

| Supplementary Table S4. Subgroup analyses for prevalence of meeting 24-Hour Movement Guidelines among U.S. adolescents aged 16-19 years, stratified by specific combinations, NHANES 2007-2016 (n= 2,273). | | | | | | | | | |
| --- | --- | --- | --- | --- | --- | --- | --- | --- | --- |
| **Subgroup items** | **Estimate U.S. population** | **Total participants, *n* (%)** | **Specific combinations, prevalence (95% CI)** | | | | | | |
|  |  |  | **Meeting only one out of three** | | |  | **Meeting any two out of three** | | |
|  |  |  | **Only meeting PA** | **Only meeting SB** | **Only meeting SLP** |  | **Meeting PA and SB** | **Meeting PA and SLP** | **Meeting SB and SLP** |
| **Gender** |  |  |  |  |  |  |  |  |  |
| Boys | 36,958,988 | 1,256 (55.3) | 14.5 (11.4,17.6) | 3.6 (2.0,5.1) | 6.7 (4.6,8.7) |  | 15.5 (12.6,18.4) | 19.5 (15.5,23.6) | 7.4 (5.3,9.5) |
| Girls | 30,475,600 | 1,017 (44.7) | 14.1 (11.2,17.0) | 8.0 (5.4,10.6) | 10.7 (8.3,13.1) |  | 11.1 (8.6,13.5) | 15.7 (11.4,20.0) | 11.7 (8.7,14.8) |
| **Race/ethnicity** |  |  |  |  |  |  |  |  |  |
| Non-Hispanic White | 39,661,366 | 690 (30.4) | 14.1 (10.8,17.4) | 5.7 (3.5,7.9) | 8.2 (5.6,10.8) |  | 12.0 (9.3,14.6) | 20.1 (15.9,24.3) | 9.0 (5.9,12.1) |
| Non-Hispanic Black | 9,631,722 | 566 (24.9) | 15.8 (12.0,19.5) | 7.1 (5.2,9.1) | 7.2 (5.1,9.3) |  | 18.9 (14.5,23.4) | 13.0 (8.9,17.2) | 9.9 (6.2,13.5) |
| Mexican American | 9,044,757 | 506 (22.3) | 10.7 (6.9,14.5) | 6.3 (3.8,8.8) | 8.7 (5.7,11.7) |  | 12.4 (8.8,16.1) | 18.7 (12.9,24.4) | 11.5 (6.6,16.4) |
| Other races (including multi-racial, other Hispanic) | 9,096,743 | 511 (22.5) | 17.3 (12.6,22.0) | 2.7 (1.1,4.2) | 11.1 (7.9,14.3) |  | 15.6 (11.4,19.9) | 11.9 (8.1,15.8) | 8.1 (5.1,11.2) |
| **PIR** |  |  |  |  |  |  |  |  |  |
| Below poverty (< 1.0) | 15,907,136 | 707 (31.1) | 11.3 (7.7,14.9) | 4.5 (2.5,6.5) | 8.1 (5.7,10.5) |  | 17.1 (13.9,20.2) | 14.7 (9.2,20.2) | 10.3 (7.3,13.4) |
| Above poverty (≥ 1.0) | 51,527,451 | 1,566 (68.9) | 15.2 (12.5,18.0) | 5.9 (4.2,7.6) | 8.6 (6.8,10.5) |  | 12.4 (10.3,14.5) | 18.7 (15.2,22.3) | 9.1 (6.7,11.4) |
| **Parental education level** |  |  |  |  |  |  |  |  |  |
| Less than 9th grade | 1,901,955 | 71 (3.1) | 12.0 (2.9,21.0) | 0.0 (0.0,0.0) | 9.2 (0.3,18.1) |  | 17.3 (2.5,32.1) | 13.0 (2.2,23.7) | 18.3 (6.8,29.7) |
| 9-12th grade (including 12th grade with no diploma) | 55,344,165 | 1,897 (83.5) | 15.9 (13.4,18.3) | 6.0 (4.3,7.7) | 8.8 (7.0,10.6) |  | 13.7 (11.6,15.9) | 18.6 (15.2,22.0) | 8.7 (6.5,10.8) |
| More than 12th grade | 10,188,467 | 305 (13.4) | 6.4 (2.6,10.3) | 4.4 (1.5,7.4) | 6.7 (3.4,10.0) |  | 11.7 (6.1,17.4) | 14.1 (8.5,19.8) | 11.5 (6.0,17.0) |
| **Total energy intake, kcal** |  |  |  |  |  |  |  |  |  |
| Quartile 1 (76,1505] | 16,626,939 | 563 (24.8) | 14.0 (9.6,18.5) | 6.1 (2.6,9.7) | 8.2 (5.9,10.4) |  | 14.7 (10.6,18.9) | 13.9 (8.7,19.0) | 13.9 (8.5,19.2) |
| Quartile 2 (1505,2014] | 15,872,385 | 568 (25.0) | 17.0 (12.7,21.4) | 7.0 (3.7,10.2) | 8.9 (5.4,12.4) |  | 9.7 (6.9,12.5) | 16.7 (11.6,21.7) | 6.1 (4.0,8.2) |
| Quartile 3 (2014,2692] | 17,927,290 | 576 (25.3) | 13.9 (10.3,17.4) | 4.2 (1.9,6.5) | 10.9 (7.9,14.0) |  | 10.6 (7.0,14.1) | 20.8 (14.8,26.8) | 9.3 (5.2,13.3) |
| Quartile 4 (2692,10436) | 17,007,973 | 566 (24.9) | 12.6 (7.7,17.4) | 5.2 (2.2,8.2) | 5.9 (2.8,9.0) |  | 19.0 (14.4,23.6) | 19.5 (14.5,24.5) | 8.1 (4.6,11.7) |
| **Survey years** |  |  |  |  |  |  |  |  |  |
| 2007-2008 | 13,862,963 | 429 (18.9) | 13.1 (8.7,17.5) | 8.8 (4.3,13.4) | 5.9 (1.9,9.9) |  | 17.3 (12.5,22.2) | 17.2 (10.8,23.6) | 8.0 (5.4,10.5) |
| 2009-2010 | 12,375,849 | 490 (21.6) | 12.5 (8.5,16.6) | 6.2 (3.6,8.8) | 7.5 (4.7,10.3) |  | 19.1 (14.7,23.6) | 14.3 (7.8,20.8) | 11.8 (8.0,15.6) |
| 2011-2012 | 13,911,006 | 423 (18.6) | 13.2 (7.5,18.8) | 3.1 (1.4,4.8) | 4.6 (2.5,6.7) |  | 11.1 (8.6,13.6) | 18.4 (10.9,26.0) | 8.5 (5.3,11.7) |
| 2013-2014 | 14,347,859 | 497 (21.9) | 16.9 (12.5,21.3) | 2.8 (0.6,4.9) | 12.2 (8.5,15.9) |  | 10.3 (5.7,15.0) | 19.6 (12.7,26.6) | 9.0 (3.2,14.7) |
| 2015-2016 | 12,936,911 | 434 (19.1) | 15.7 (9.2,22.2) | 7.3 (4.2,10.4) | 12.4 (8.0,16.8) |  | 10.3 (7.0,13.5) | 19.0 (11.7,26.2) | 9.9 (5.4,14.5) |
| Footnotes: Data are presented as survey-weighted prevalence (%) and 95% CI. CI, confidence interval; NHANES, National Health and Nutrition Examination Survey; PA, physical activity; PIR, poverty to income ratio; SB, sedentary behavior; SLP, sleep. | | | | | | | | | |
